# Radiologische Manifestationen von Lungenerkrankungen bei COVID-19

**DOI:** 10.1007/s00117-020-00749-4

**Published:** 2020-09-08

**Authors:** Benedikt H. Heidinger, Daria Kifjak, Florian Prayer, Lucian Beer, Ruxandra-Iulia Milos, Sebastian Röhrich, Hanka Arndt, Helmut Prosch

**Affiliations:** 1 Universitätsklinik für Radiologie und Nuklearmedizin, Medizinische Universität Wien, Währinger Gürtel 18–20, 1090 Wien, Österreich; 2 Institut für Diagnostische und Interventionelle Radiologie, Kinder- und Neuroradiologie, Universitätsmedizin Rostock, Rostock, Deutschland

**Keywords:** Computertomographie, Differenzialdiagnosen, Milchglasverdichtung, Konsolidierungsareale, Fibrotische Lungenveränderungen, Computed tomography, Differential diagnosis, Ground glass opacifications, Areas of consolidation, Fibrotic lung parenchyma

## Abstract

**Klinisches/methodisches Problem:**

Seit dem Auftreten des neuartigen Coronavirus Ende 2019 und der damit verbundenen Erkrankung – Coronavirus Disease 2019 (COVID-19) – kam es zum Ausrufen einer Pandemie durch die Weltgesundheitsorganisation (WHO). Der Referenzstandard für die Diagnose ist der Virusnachweis mittels „reverse transcription polymerase chain reaction“ (RT-PCR). Bei hoher Spezifizität ist die Sensitivität der RT-PCR jedoch stark abhängig von der Symptomdauer, der Viruslast, der Qualität der Probe sowie des verwendeten Tests.

**Radiologische Standardverfahren:**

Im Rahmen von COVID-19 werden primär Thoraxröntgen und Thorax-Computertomographie(CT) zur Erkennung von Lungenmanifestationen bzw. deren Ausdehnung und von Komplikationen eingesetzt.

**Leistungsfähigkeit:**

Die Sensitivität und Spezifizität des Thoraxröntgens bei COVID-19 ist gering. Die Thorax-CT weist eine hohe Sensitivität von ungefähr 90 % bei jedoch geringer Spezifizität auf (zwischen 25 und 33 %).

**Empfehlung für die Praxis:**

Die Indikation für die Durchführung von Bildgebung im Rahmen von COVID-19 sollte immer mit Bedacht gestellt werden, um das Übertragungsrisiko für medizinisches Personal und andere Patienten zu minimieren. Die Bildgebung ist vor allem hilfreich zur Evaluierung des Ausmaßes der Lungenbeteiligung der Erkrankung, zur Abgrenzung von Komplikationen und Differenzialdiagnosen. Typischerweise zeigen sich bilaterale, subpleurale Milchglasverdichtungen mit oder ohne Konsolidierungsareale. Im Verlauf können auch Veränderungen einer organisierenden Pneumonie beobachtet werden. Bei Untersuchungen nach Genesung einer COVID-19-Pneumonie ist auf fibrotische Lungenveränderungen zu achten.

## Abstract (en)

**Clinical issue:**

Since its emergence in late 2019, the disease caused by the novel coronavirus, termed COVID-19, has been declared a pandemic by the World Health Organization. Reference standard for the diagnosis of COVID-19 is a positive reverse transcription polymerase chain reaction (RT-PCR) test. While the RT-PCR shows a high specificity, its sensitivity depends on the duration of symptoms, viral load, quality of the sample, and the assay used.

**Standard radiological methods:**

Chest radiography and computed tomography (CT) of the chest are the imaging modalities primarily used for assessment of the lung manifestations, extent, and complications of COVID-19 pneumonia.

**Performance:**

Sensitivity and specificity of chest radiography is low. While sensitivity of CT for detecting COVID-19 pneumonia is high—averaging around 90%—its specificity is low—between 25 and 33%.

**Practical recommendations:**

Indications for imaging in patients with suspected or diagnosed COVID-19 infection should be carefully considered to minimize the risk of infection for medical personnel and other patients. Imaging, particularly CT, can assess disease extent, complications, and differential diagnoses. COVID-19 pneumonia typically presents with bilateral, subpleural areas of ground glass opacifications with or without consolidations. During the course of the disease features resembling organizing pneumonia can occur. Follow-up examinations after recovery from COVID-19 pneumonia should focus on fibrotic changes of the lung parenchyma.

## Hintergrund

Seit der erstmaligen Beschreibung des neuartigen Coronavirus, genannt „severe acute respiratory syndrome coronavirus 2“ (SARS-CoV-2), Ende 2019 in China hat sich das Virus global ausgebreitet mit weltweit über 17 Mio. gemeldeten positiven Fällen und mehr als 650.000 an der Infektion mit SARS-CoV‑2 verstorbenen Patienten bis Ende Juli 2020 [1]. Die durch SARS-CoV‑2 hervorgerufene Erkrankung wird unter dem Namen „COronaVIrus Disease 2019“ (COVID-19) zusammengefasst. SARS-CoV‑2 gehört zur selben Gruppe von Erregern, die 2002/2003 für das „severe acute respiratory syndrome“ (SARS) im asiatischen Raum verantwortlich war sowie für das „middle east respiratory syndrome“ (MERS), welches seit 2013 immer wieder im Mittleren Osten auftritt.

## Klinik und Laborveränderungen

Wie im Namen des Virus – „severe acute respiratory syndrome“ – bereits angedeutet, manifestiert sich COVID-19 überwiegend mit respiratorischen Symptomen [2]. Trockener Husten und Dyspnoe stehen hier neben Fieber im Vordergrund [2], wobei die Infektion häufig auch einen asymptomatischen Verlauf nehmen kann [3]. COVID-19 kann sich aber auch mit gastrointestinalen Symptomen, z. B. Durchfall, oder Anosmie oder Geschmacksverlust manifestieren [2].

Laborchemisch zeigt sich oftmals, ähnlich wie bei anderen viralen pulmonalen Erkrankungen, eine unspezifische Lymphozytopenie und/oder Leukozytopenie, wobei auch eine Leukozytose vorkommen kann. Weitere Veränderungen beinhalten eine Thrombozytopenie, erhöhte CRP(C-reaktives Protein)- sowie LDH(Laktatdehydrogenase)-Werte und erhöhtes D‑Dimer [2].

## Diagnose

Der Referenzstandard für die Diagnose von COVID-19 ist eine positive „reverse transcription polymerase chain reaction“ (RT-PCR) eines Nasen‑/Rachenabstriches oder einer Probe tiefen Bronchialsekrets [4]. Die RT-PCR weist eine sehr hohe Spezifizität auf [4]. Die Sensitivität der RT-PCR liegt bei 89 % mit einer Schwankungsbreite von 60–98 % und ist stark abhängig von der Symptomdauer, der Viruslast, der Qualität der Probe sowie des verwendeten Tests [4].

## Indikationen für die Lungenbildgebung

Mehrere medizinische und radiologische Fachgesellschaften haben Empfehlungen für die Anwendung der verschiedenen Bildgebungsmodalitäten bei Patienten mit Verdacht auf oder bereits nachgewiesener SARS-CoV‑2 publiziert [5–10]. Es besteht eine generelle Übereinstimmung, dass die Indikationen für die Bildgebung einheitlich angewandt und die Bildgebung mit Bedacht eingesetzt werden sollte, um das Ansteckungsrisiko für das medizinische Personal und andere Patienten zu minimieren. Bei asymptomatischen Patienten und bei Patienten mit nur geringen Symptomen sollte daher keine Lungenbildgebung erfolgen [5–10].

Thoraxröntgenuntersuchungen sind weithin verfügbar, können am Patientenbett durchgeführt werden und gehen im Vergleich zur Computertomographie (CT) mit einer geringeren Strahlendosis einher [11]. Dadurch wird das transportbedingte Ansteckungsrisiko für das medizinische Personal und andere Patienten minimiert [8]. Sensitivität und Spezifizität des Thoraxröntgens sind, insbesondere bei früher oder milder SARS-Cov2-Infektion, gering [8]. Beispielsweise zeigten in einer Studie lediglich 69 % der Aufnahmeröntgenbilder bei SARS-CoV2-RT-PCR-positiven Patienten Veränderungen [12]. Somit ist das Thoraxröntgen zur Diagnose von COVID-19 nur eingeschränkt geeignet [7].

Bei gesicherten COVID-19-Fällen kann ein Thoraxröntgen für die Beurteilung einer klinischen Verschlechterung oder von Komplikationen wie z. B. von Pleuraergüssen eingesetzt werden [8]. Tägliche (Routine‑)Verlaufskontrollen mittels Thoraxröntgen bei stabilen Patienten, auch wenn diese intubiert sind, sind nicht indiziert [8].

Die Indikation zur Durchführung einer Thorax-CT bei Patienten mit Verdacht auf oder bereits diagnostizierter Covid-19-Pneumonie sollte immer mit Bedacht gestellt werden. Die Durchführung einer CT ist aufgrund des Transports an die radiologische Abteilung mit einem erhöhten Ansteckungsrisiko für das medizinische Personal und andere Patienten verbunden [8]. Die CT hat eine hohe Sensitivität von 92 % für die Detektion von Lungenmanifestationen im Rahmen von COVID-19 bei gleichzeitig geringer Spezifizität, welche zwischen 25 und 33 % liegt [13, 14]. Aufgrund der deutlich höheren Spezifizität beruht die Diagnose einer SARS-CoV2-Infektion prinzipiell auf einer positiven RT-PCR [6–8].

Bei bereits diagnostizierten Patienten mit mäßiger, schwerer oder progredienter Symptomatik bzw. mit einem hohen Risiko auf einen schweren Krankheitsverlauf – z. B. Patienten mit Diabetes, Autoimmunerkrankungen, Adipositas oder vorbekannter Lungenerkrankung – kann eine CT in der initialen Phase und zur Verlaufsbeurteilung durchgeführt werden [7, 8]. Bei diesen Patienten kann die CT zur Abschätzung der Ausdehnung der Erkrankung, zur Diagnose von Komplikationen oder zusätzlicher zugrunde liegender Erkrankungen hilfreich sein [7, 8, 10]. Bei fortbestehender Beeinträchtigung der Lungenfunktion oder Hypoxämie nach Genesung von COVID-19 ist eine CT ebenso indiziert [8]. Nicht notwendig ist die Durchführung einer CT bei klinischer Besserung der Beschwerden bzw. ohne fortbestehende Beeinträchtigung [8, 9].

Sollten sich COVID-19-typische Lungenveränderungen als Zufallsbefund bei respiratorisch asymptomatischen Patienten zeigen, ist eine Bestätigung der Diagnose mittels RT-PCR notwendig [7, 8]. Bei Patienten mit COVID-19-typischen Lungenveränderungen, jedoch negativer RT-PCR, sollte die RT-PCR zum Ausschluss eines falsch-negativen Ergebnisses wiederholt werden [7, 10].

Prinzipiell ist für die Beurteilung der Lungenbeteiligung eine native Thorax-CT in tiefer Inspirationslage geeignet [7]. Besteht jedoch der Verdacht auf eine im Rahmen von COVID-19 gehäuft auftretende Pulmonalembolie, sollte eine kontrastmittelunterstützte CT in pulmonalarterieller Phase durchgeführt werden [7]. Falls vorhanden, sollte in solchen Fällen außerdem eine Evaluierung mittels Dual-energy-CT in Betracht gezogen werden, um kleine, durch mikrovaskuläre Thromben verursachte Perfusionsdefizite nachzuweisen [15, 16].

## Radiologische Manifestationen

### Lungenultraschall

Der Einsatz von Lungenultraschall im Rahmen von COVID-19 sowie deren Manifestationen wird in einem gesonderten Artikel in diesem Heft detailliert diskutiert [17].

### Thoraxröntgen

**Figure 1:**
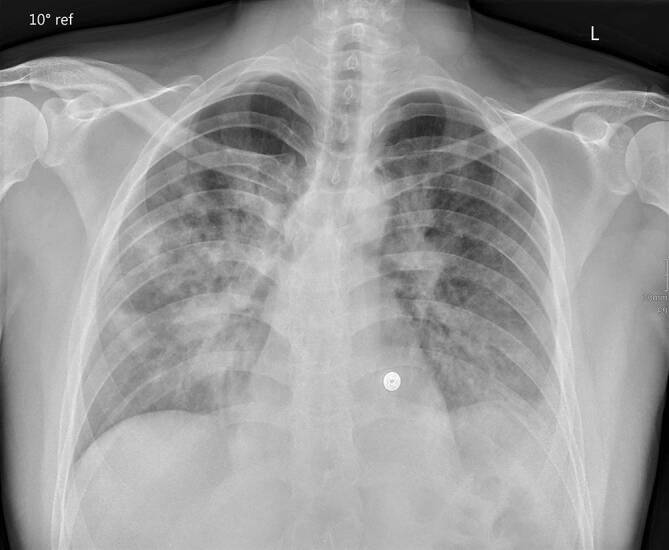

Im Thoraxröntgen manifestiert sich COVID-19 am häufigsten mit Milchglasverdichtungen und/oder Konsolidierungsarealen [12, 18, 19]. Typischerweise zeigen sich diese Veränderungen bilateral, multifokal mit einer peripheren Verteilung und einer geringen Prädominanz der Unterfelder (Abb. 1; [12, 18, 19]). Diffuse Parenchymverdichtungen im Thoraxröntgenbild im Rahmen von COVID-19 können Ausdruck eines akuten Lungenversagens („acute respiratory distress syndrome“, ARDS) sein [19]. Im Thoraxröntgen untypisch für COVID-19 sind Kavitationen und Pleuraergüsse, die hinweisend auf Komplikationen oder andere Diagnosen wie beispielsweise eine kardiale Dekompensation sein können [19].

### Computertomographie des Thorax

Die Veränderungen des Lungenparenchyms, die sich im Rahmen einer COVID-19-Pneumonie in der CT zeigen, sind unspezifisch und oft schwer von anderen Diagnosen zu unterscheiden. Besteht jedoch eine hohe klinische Vortestwahrscheinlichkeit für COVID-19, beispielsweise bei typischen klinischen Symptomen und bekanntem Kontakt zu einer SARS-CoV2-positiven Person oder einer hohen Erkrankungsprävalenz in der Bevölkerung, sind diese jedoch als wahrscheinlich für das Vorliegen einer COVID-19-Pneumonie zu werten. Die Diagnose muss jedoch immer mittels RT-PCR gesichert werden [8].

**Figure 2:**
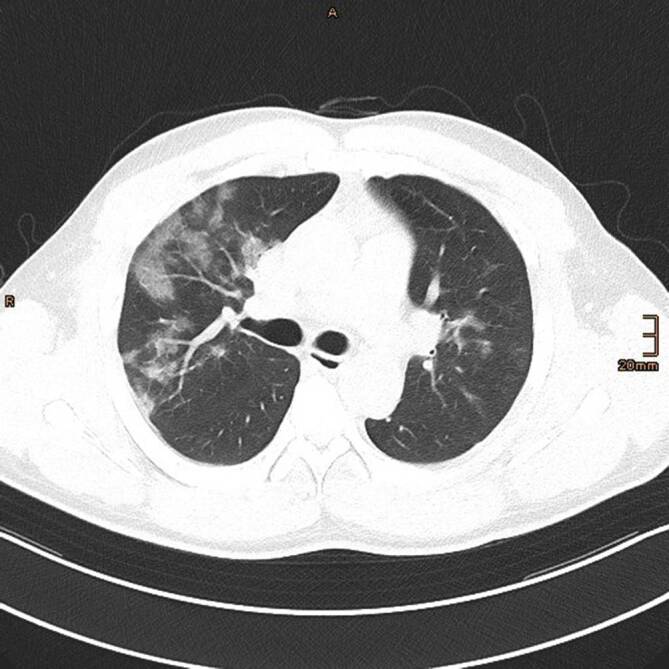

In der Thorax-CT zeigen sich initial am häufigsten Milchglasverdichtungen mit oder ohne Konsolidierungsareale (Abb. 2, 3 und 4; [20, 21]). Konsolidierungsareale können auch ohne Milchglasverdichtungen vorkommen oder ein zentrales Milchglasareal im Sinne eines „umgekehrtes Halo-Zeichen“ umgeben [20, 21]. Gelegentlich zeigen sich verdickte Interlobärsepten, teilweise gemeinsam mit Milchglas („crazy paving“; [20]). Die Lungenparenchymveränderungen treten typischerweise bilateral, multifokal und in einer subpleuralen bzw. peripheren Verteilung auf, wobei oft eine (geringe) Prädominanz basaler und dorsaler Lungenabschnitte vorliegt [20, 21]. Die Veränderungen reichen von kleinen, rundlichen Arealen bis zu großflächigen Parenchymverdichtungen [20, 21]. Für COVID-19 nichttypische Manifestationen inkludieren Lymphadenopathie, Kavitationen oder noduläre Verteilungsmuster wie Tree-in-bud-Noduli [20, 21]. Pleuraergüsse sind selten [20, 21]. Letztere Veränderungen sind oft hinweisend auf das Vorliegen einer anderen Erkrankung oder einer Komplikation im Rahmen einer COVID-19-Pneumonie, wie z. B. eine kardiale Mitbeteiligung oder eine bakterielle Pneumonie.

**Figure 3:**
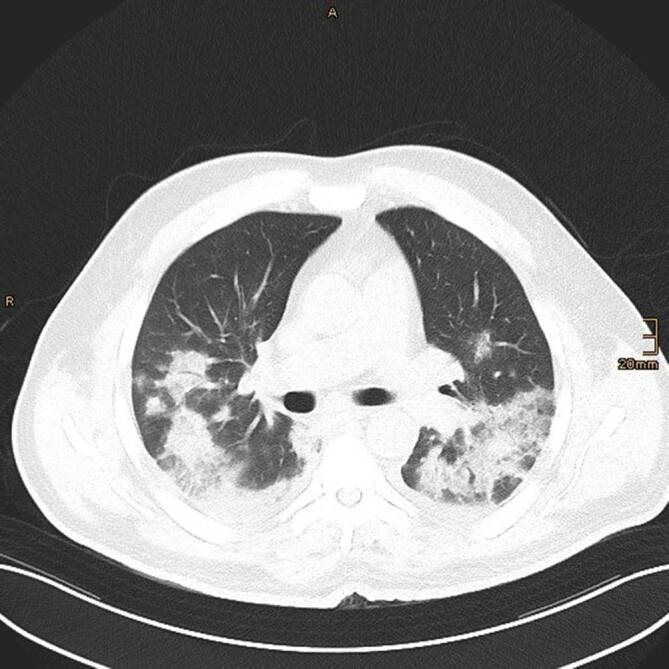

**Figure 4:**
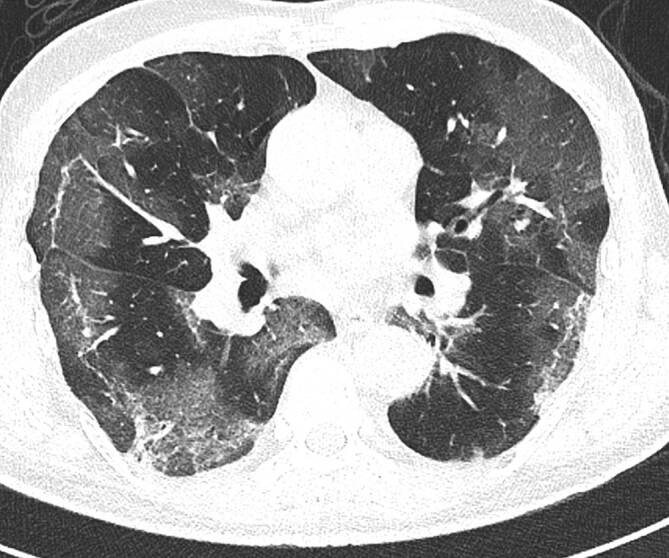

Während initial typischerweise fokale oder multifokale Milchglasveränderungen zu beobachten sind, nimmt die Ausdehnung und die Dichte der Lungenparenchymveränderungen im Krankheitsverlauf zu [21–26]. So treten Konsolidierungsareale im Verlauf häufiger auf als im Initialstadium [22–24, 26]. Die maximale Ausprägung der Lungenbeteiligung wird zumeist zwischen dem 9. und 13. Tag nach Symptombeginn erreicht [21, 27, 28]. Danach sind die Dichte und Ausdehnung der Lungenparenchymveränderungen bei positivem Verlauf der Erkrankung rückläufig [22–24, 26].

**Figure 5:**
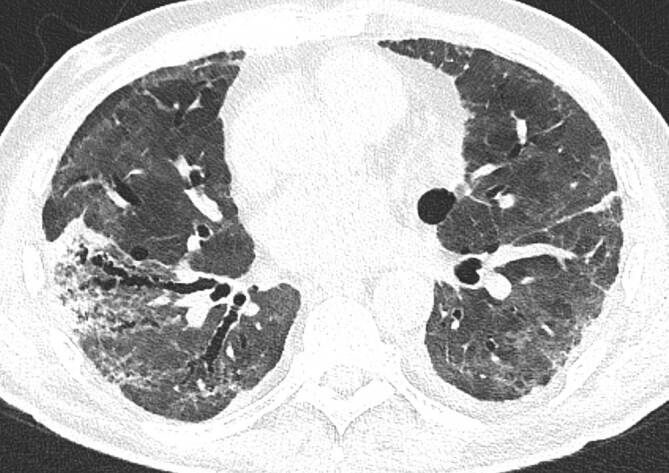

Im weiteren Verlauf kann es zur Bildung strangförmiger, arkadenartiger, subpleuraler Verdichtungen kommen, die als „fibrous stripes“ bezeichnet werden und histologisch meist einer organisierenden Pneumonie entsprechen (Abb. 5; [21]). Mit einer vollständigen Regredienz der Veränderungen ist frühestens nach 25 Tagen zu rechnen. Bei manchen Patienten sind Lungenparenchymveränderungen wie flaue Milchglasareale, „crazy paving“ oder interstitielle Veränderungen noch drei Monate nach wiederholt negativer SARS-CoV2-RT-PCR CT-morphologisch nachweisbar [29].

## Radiologische Manifestationen von Komplikationen

### Bakterielle Infektion

An das Vorliegen einer zusätzlichen bakteriellen Infektion ist beim Vorliegen einer Lymphadenopathie und von Pleuraergüssen zu denken. Auch Tree-in-bud-Noduli und Kavitationen deuten auf eine bakterielle Infektion hin [11].

### Akutes Lungenversagen

Die schwerste Form der Lungenbeteiligung im Rahmen von COVID-19 manifestiert sich klinisch als akutes Lungenversagen [30]. Radiologisch zeigen sich hierbei typischerweise beidseits diffus verteilte ausgeprägte Milchglasverdichtungen und/oder Konsolidierungsareale [11].

### Kardiale Mitbeteiligung

Im Rahmen von SARS-CoV-2-Infektionen kann es in bis zu 25 % der Fälle zu einer Schädigung des Myokards, insbesondere einer Myokarditis, oder einer Herzinsuffizienz kommen [31], wobei bei fast 80 % der Patienten kurz nach der Genesung kardiale Veränderungen in der Magnetresonanztomographie (MRT) nachweisbar waren [32]. Indirekte Hinweise auf eine kardiale Mitbeteiligung in der CT können Zeichen eines Lungenödems sowie das Auftreten von Pleuraergüssen sein [7]. Diese Veränderungen sollten mit Troponinwerten und elektrokardiographischen (EKG-)Veränderungen korreliert sowie eine Echokardiographie durchgeführt werden [7]>

### Pulmonalembolie/intraarterielle Thrombose

Eine Pulmonalembolie ist eine häufige Komplikation im Rahmen von COVID-19 und wird in über 35 % der CT-Angiographien nachgewiesen [33]. In einer pathologischen Fallserie konnten bei allen an COVID-19 verstorbenen Patienten Thrombosen in kleinen und mittelgroßen Pulmonalarterien nachgewiesen werden [34]. In einer weiteren pathologischen Fallserie verstarb ein Drittel der Patienten an einer massiven Pulmonalembolie, ausgelöst durch eine bilaterale tiefe Beinvenenthrombose [35]. Auch wenn der Nachweis zeitgleicher tiefer Beinvenenthrombosen in der letztgenannten Fallserie für ein venöses thromboembolisches Geschehen spricht, ist es noch unklar, ob es sich hierbei tatsächlich um ein venöses thromboembolisches Geschehen, eine arterielle intravaskuläre Thrombose oder eine Kombination beider Entitäten handelt [16, 34–36].

Für eine intravaskuläre Thrombose würde sprechen, dass die Gefäße vollständig okkludiert und kleinere Pulmonalarterien mit einem Durchmesser von weniger als einem Millimeter mitbetroffen waren [34]. Diese Veränderungen stehen wahrscheinlich im Zusammenhang mit der Entzündungsreaktion bzw. mit den Reparaturmechanismen des durch die Infektion ausgelösten diffusen Alveolarschadens [34, 37]. Des Weiteren verursacht das Virus einen Endothelschaden, der prothrombotische Wirkung hat [34, 37].

Besteht der klinische Verdacht auf eine Pulmonalembolie bzw. eine intraarterielle Thrombose, sollte eine kontrastmittelgestützte CT durchgeführt werden, idealerweise mit einem Dual-energy-Protokoll, um auch kleinere Perfusionsdefizite nachweisen zu können [7, 15].

### Barotrauma

Bei intubierten und mechanisch beatmeten Patienten ist besonders auf das Auftreten von Barotraumata zu achten. So treten Pneumothorax, Pneumomediastinum und Pneumoperikard bei ca. 15 % aller beatmeten COVID-19-Patienten und somit deutlich häufiger als in einer vergleichbaren SARS-CoV2-negativen Kontrollgruppe auf [38].

### Fibrotische Lungenveränderungen

Bei MERS und SARS kam es nach Abheilung der Infektion bei bis zu 30 % der Patienten zu fibrotischen Lungenveränderungen [39]. Wie hoch die Prävalenz solcher Veränderungen bei COVID-19 ist, ist derzeit noch nicht bekannt. Es gibt jedoch bereits Berichte über Patienten, die nach der Genesung von COVID-19 fibrotische Lungenveränderungen aufweisen ([29]; Abb. 5). Aufgrund dessen ist bei der Befundung einer CT nach einer Genesung von COVID-19 insbesondere auf fibrotische Lungenveränderungen wie Traktionsbronchiektasien, „honeycombing“ oder Retikulationen zu achten.

## Radiologische Manifestationen von Differenzialdiagnosen

Aufgrund des letztendlich unspezifischen Erscheinungsbildes in der CT kommen differenzialdiagnostisch neben anderen viralen Pneumonien, z. B. verursacht durch Influenzaviren, Adenoviren und SARS, bakterielle Pneumonien infrage. Des Weiteren ist differenzialdiagnostisch an nichtinfektiöse Ursachen wie Herzinsuffizienz, organisierende Pneumonie anderer Ätiologie sowie an eosinophile Pneumonie zu denken [11, 40].

**Figure 6:**
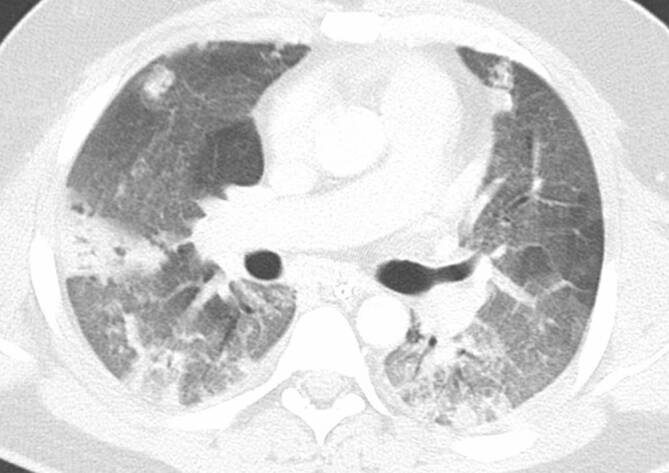

Pneumonien, welche durch das Influenzavirus verursacht werden, manifestieren sich CT-morphologisch meist als unilaterale oder bilaterale Milchglasverdichtungen mit multifokalen Konsolidierungen in bevorzugt subpleuraler und peribronchovaskulärer Verteilung und gelegentlich mit einem „Crazy-paving“-Muster (Abb. 6; [41]). Adenoviruspneumonien präsentieren sich überwiegend bei Kindern als multifokale, Milchglasverdichtungen mit fleckigen Konsolidierungsarealen sowie mit Atelektasen, die bevorzugt im rechten Oberlappen auftreten [11, 41]. Bei MERS zeigen sich bilaterale, periphere, fokale oder multifokale Milchglasverdichtungen und Konsolidierungen [11]. Aufgrund der überlappenden Muster in der CT-Bildgebung ist eine Unterscheidung zur COVID-19-Pneumonie oft unmöglich [40].

Eine bakterielle Pneumonie kann oft anhand von Konsolidierungen, die typischerweise auf ein Segment oder einen Lappen begrenzt sind, von einer COVID-19-Pneumonie unterschieden werden. Zusätzlich können bronchiale Wandverdickungen, zentrilobuläre Noduli, bronchialer Sekretstau, Phänomene die bei einer COVID-19-Pneumonie selten sind, auftreten [40].

**Figure 7:**
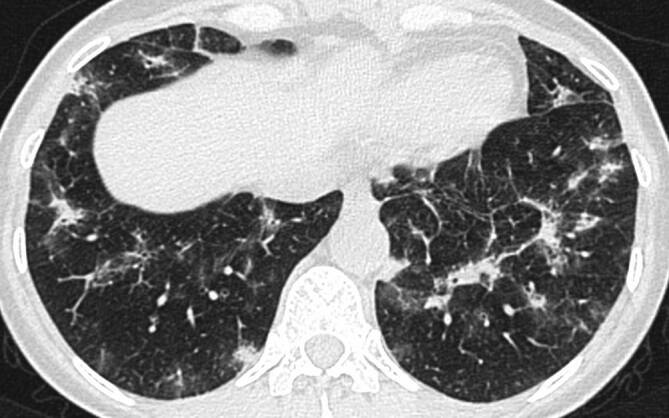

Bei der organisierenden Pneumonie (OP) – als Zeichen einer nichtspezifischen Gewebsantwort auf eine Verletzung des Lungenparenchyms – stehen CT-morphologisch meist bilaterale, strangförmige oder peripher betonte arkadenartige Verdichtungen oder auch fokale, irreguläre Konsolidierungsareale im Vordergrund. Diese zeigen eine peribronchovaskuläre oder subpleurale Verteilung. Des Weiteren finden sich gelegentlich Milchglasverdichtungen, Bronchiektasen sowie Architekturstörungen des Lungenparenchyms (Abb. 7; [42]).

**Figure 8:**
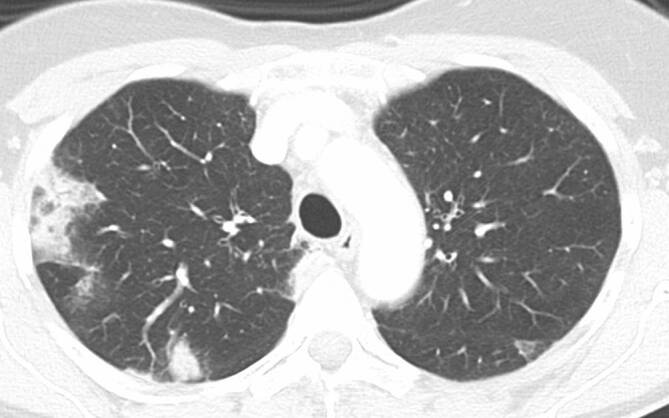

Die akute eosinophile Pneumonie präsentiert sich in der Bildgebung bilateral mit fleckigen Milchglasverdichtungen, welche häufig von verdickten interlobulären Septen, manchmal von Konsolidierungen oder unscharf definierten Knötchen begleitet werden. Gelegentlich zeigen sich Pleuraergüsse [43]. Die chronische eosinophile Pneumonie manifestiert sich in der Bildgebung mit nichtsegmentalen Konsolidierungen mit peripherer Prädominanz (Abb. 8; [43]).

Das kardiogene Lungenödem, verursacht durch eine Linksherzinsuffizienz, zeigt sich in der CT mit verdickten interlobulären Septen und Bronchialwänden als Zeichen eines interstitiellen Lungenödems. Des Weiteren finden sich symmetrische, teils multiple oder diffuse Milchglasverdichtungen und Konsolidierungen als radiologische Manifestationen eines alveolären Lungenödems [44]. Zusätzlich sind ein vergrößertes Herz und bilaterale Pleuraergüsse hinweisend auf ein kardiogenes Lungenödem [44].

## Systematische Befundung von Patienten mit COVID-19

Bei der Befundung von CT-Bildern von Patienten mit Verdacht auf COVID-19 sollte auf die Wahrscheinlichkeit des Bestehens einer COVID-19-Pneumonie eingegangen werden. Hierbei können anhand der oben beschriebenen Lungenveränderungen die folgenden drei Kategorien unterschieden werden:

1. COVID-19 wahrscheinlich,
2. COVID-19 nicht auszuschließen,
3. keine COVID-19-typischen Veränderungen [7, 10, 45].

Die erste Kategorie umfasst Lungenveränderungen wie periphere Milchglasverdichtungen, die typisch für eine COVID-19-Pneumonie sind. In die zweite Kategorie fallen all jene Patienten, bei denen die Veränderungen im Rahmen einer COVID-19-Pneumonie auftreten können, jedoch andere Diagnosen ebenso wahrscheinlich sind. In diesem Fall sollten die entsprechenden Differenzialdiagnosen im Befund angeführt werden. Die letzte Kategorie beinhaltet einerseits pulmonale Veränderungen, die klar einer anderen Diagnose zugeordnet werden können bzw. mit COVID-19 nicht vereinbar sind, andererseits ein unauffälliger Lungenbefund. Sollten die Veränderungen anderen Diagnosen zuordenbar sein, sollten diese im Befund benannt werden.

Ein Befundschema (modifiziert nach Revel et al.) für das Lungenparenchym in einer nativen CT des Thorax bei Patienten mit Verdacht auf COVID-19 oder bereits diagnostiziertem COVID-19 ist in Tab. 1 zu finden [7].

**Table 1:** 

*Befund*
(Unilaterale/bilaterale, diffuse, konfluierende, multifokale, rundliche) Milchglasverdichtungen mit einem „Crazy-paving“-Muster/peripherer Verteilung ohne subpleurale Aussparungen
Milchglasverdichtungen und perilobuläre Konsolidierungsareale/lineare/arkadenförmige Konsolidierungsareale
(Kein) Vorliegen eines Tree-in-bud Musters/zentrilobulärer Knötchen/endobronchialen Sektretstaus/lobärer oder segmentaler Konsolidierungsareale
(Kein) Vorliegen einer Lymphadenopathie/von Pleuraergüssen
*Zusammenfassung*
Lungenparenchymveränderungen, die charakteristisch für eine COVID-19-Pneumonie mit geringer/mäßiger/schwerer Ausprägung^a^ sind
Lungenparenchymveränderungen, bei denen ein viral-entzündliches Geschehen und somit COVID-19 möglich, jedoch nicht charakteristisch ist
Lungenparenchymveränderungen, die nicht charakteristisch für COVID-19 sind
Andere Strukturen, wie das Herz und die Gefäße, die Pleura, das Mediastinum, die Knochen und die Weichteile sollten wie gewohnt befundet werden

^a^Basierend auf einer visuellen Graduierung. Eine Skala (z. B.: <10 %, 10–25 %, 25–50 %, 50–75 %, >75 %) kann hier verwendet werden

## Fazit für die Praxis

- Die Bildgebung spielt im Rahmen der COVID-19-Pandemie eine wichtige Rolle. Sie sollte allerdings immer mit Bedacht eingesetzt werden, um das Ansteckungsrisiko für das Personal und andere Patienten zu minimieren.
- In der Computertomographie (CT) zeigen sich typischerweise bilaterale, subpleurale Milchglasverdichtungen mit oder ohne Konsolidierungsareale. In Verlauf können auch Parenchymveränderungen einer organisierenden Pneumonie beobachtet werden. Residuäre Veränderungen können bei einigen Patienten noch Monate nach Abklingen der Symptomatik nachweisbar sein. Gelegentlich zeigen sich im Verlauf fibrotische Lungenveränderungen.
- Die kontrastmittelunterstützte CT, vor allem unter Anwendung der Dual-energy-Technologie, kann Perfusionsdefizite im Zusammenhang mit Pulmonalembolien oder intraarterieller Gerinnung nachweisen.
